# Differences between remote and analog design thinking through the lens of distributed cognition

**DOI:** 10.3389/frai.2022.915922

**Published:** 2022-11-17

**Authors:** Daniel Wolferts, Elisabeth Stein, Ann-Kathrin Bernards, René Reiners

**Affiliations:** ^1^Department for Human-Centered Engineering and Design, Fraunhofer Institute for Applied Information Technology, Sankt Augustin, Germany; ^2^Department for Data Science and AI, Fraunhofer Institute for Applied Information Technology, Sankt Augustin, Germany

**Keywords:** human-computer interaction (HCI), artificial intelligence (AI), distributed cognition for teamwork, Design Thinking (DT), remote work

## Abstract

Due to the huge surge in remote work all over the world caused by the COVID-19 pandemic, today's work is largely defined by tools for information exchange as well as new complex problems that must be solved. Design Thinking offers a well-known and established methodological approach for iterative, collaborative and interdisciplinary problem solving. Still, recent circumstances shed a new light on how to facilitate Design Thinking activities in a remote rather than an analog way. Due to Design Thinking's high production of artifacts and its focus on communication and interaction between team members, the theory of Distributed Cognition, specifically the Distributed Cognition for Teamwork (DiCoT) framework, provides an interesting perspective on the recent going-remote of Design Thinking activities. For this, we first highlight differences of analog vs. remote Design Thinking by analyzing corresponding literature from the recent years. Next, we apply the DiCoT framework to those findings, pointing out implications for practical facilitation of Design Thinking activities in an analog and remote setting. Finally, we discuss opportunities through artificial intelligence-based technologies and methods.

## 1. Introduction

In recent years, due to the COVID-19 pandemic, the world experienced a spike in new digital work and new ways of learning (Brynjolfsson et al., 2020; De' et al., 2020; Feldmann et al., 2021). A lot of professional collaboration between individuals, as well as their interaction with work tools, have become digitized, which affects their work environment and thus results in behavior change within teams. This includes video conferencing tools that have become the go-to mode of communication in team meetings and digital whiteboards or other collaborative software tools that support creativity and productivity (Unger et al., 2021). With industries shifting their focus from production to more service oriented knowledge work, the need for innovative solutions has been growing constantly (Brown, 2008; Kane et al., 2018). For this purpose many industries have started thinking outside the box and turning to previously unfamiliar disciplines to find new ways of innovative working and problem solving. Design is one of those disciplines and *Design Thinking* (from here on referred to as *DT*) has become a popular framework to facilitate the creation of innovation and to find new solutions to complex, so called wicked problems (Buchanan, 1992). Wicked problems are those type of problems that have, among other attributes, (a) no definitive predefined problem formulation, (b) no stopping rule (i.e., the problem solver can always do better), and (c) no definitive right-or-wrong solution criteria catalog (Kunz and Rittel, 1972). With the ongoing digitization of tools and artifacts, such as collaboration platforms or design tools, these supporting technologies are also becoming “smarter.” For instance, Suleri et al. (2019) have introduced an Artificial Intelligence (AI)-powered prototyping tool that lets designers create low-fidelity prototypes and evolves them into mid- to high-fidelity design drafts. Add to this the current trend of going-remote and related discussions of a “post-pandemic workplace” (Kane et al., 2021), we find it necessary to examine the implications of these technological trends on DT practices. The aim of our study is twofold: We first want to highlight and examine differences between analog and remote DT practices. Second, we want to assess the applicability of Distributed Cognition as a guiding theory for researching DT practices in both, analog and remote settings. For this reason, we look at DT and what the going-remote means for DT practices and practitioners. Due to DT's high production of artifacts and its focus on communication and interaction between team members, we use the theory of Distributed Cognition (DCog) as a lens to examine how interactions in the remote differ from interactions in the analog world. Authors like Blandford and Furniss (2005), Webb (2008), or Deshpande et al. (2016) have already looked at Distributed Cognition as a theory to inform research on collaboration in (agile) teams. Our research expands on this notion by examining collaboration in DT, specifically when conducted remotely, and what this means for Design Thinkers' interaction with artifacts, as well as with other individuals. We conclude by alluding to the role of AI in these interactions and highlighting the limitations of this paper as well as future research directions.

## 2. Background

In this section we present the underlying concepts behind DT and DCog. After that, we introduce our methodological approach to examine the differences between remote and analog DT with respect to DCog.

### 2.1. Design thinking

DT, despite its growing popularity, is not a clearly defined and universally agreed upon concept. It rather serves as an umbrella term for a diverse conglomerate of *understandings* about human-centered, agile, multi-disciplinary, and creative ways of creating new solutions to existing problems. Scientific literature reveals several attempts to describe attributes common to the different DT approaches.

In its origins, DT has largely been defined by the work of designers (see e.g., Brown 2008), but has now become a multi-disciplinary framework—or as Lindberg et al. (2010, p. 35) put it: “Design thinking understood as a meta-disciplinary methodology loosens the link to design as a profession.” Accordingly, DT has increasingly found its way into several research and application domains and practitioners and researchers from different fields of application have started to embrace the “designerly ways of knowing” (Cross, 1982). For example, Kimbell (2011, p. 295) suggests that DT de-politizises managerial practice, in that it helps managers to “shift from choosing between alternatives to helping them generate entirely new concepts.” DT has also sparked the interest of psychologists in terms of individual behavior and group dynamics. In this vein, Liedtka (2015) point to DT's potential to reduce cognitive biases in decision making, for example through methodologies that are innate to DT, like perspective-taking, working in teams, or a strong reliance on empirical evidence. Roberts et al. (2016) examine DT's potential for health care, in that it can help health-care professionals to find solutions to complex and overarching problems, like the increase in diabetes and obesity, and help them to bridge the gap between abstract and high-level issues and physicians' day-to-day work. Depiné et al. (2017) describe the integrative nature of DT in their study about Smart Cities. They identified DT's potential not just for developing new technological solutions, but also for integrating citizens needs and concerns as an integral part of the process.

Brenner et al. (2016) understand DT as a triad of *mindset, process*, and *toolbox*. With *mindset* they describe a number of guiding principles that Design Thinkers follow, like human-centeredness, applying divergent and convergent thinking, early prototyping, and creating the right environment for creative problem solving. As for *process*, they define an iterative five-step loop of activities regarding problem definition, need-finding and synthesis, ideate (i.e., idea brainstorming), prototyping, and testing. Lastly, they describe a number of tools and methods as the *toolbox* of Design Thinkers, like observation, storytelling, personas, and empathy maps. Its lack of a clear definition, however, can be considered being part of it strengths, because it allows DT “to be the right thing at the right time” (Zimmerman et al., 2007, p. 494). Brown (2008, p. 1) calls DT “a methodology that imbues the full spectrum of innovation activities with a human-centered design ethos.” It can be described as a “system of spaces,” in which different types of activities take place.

**Figure 1:**
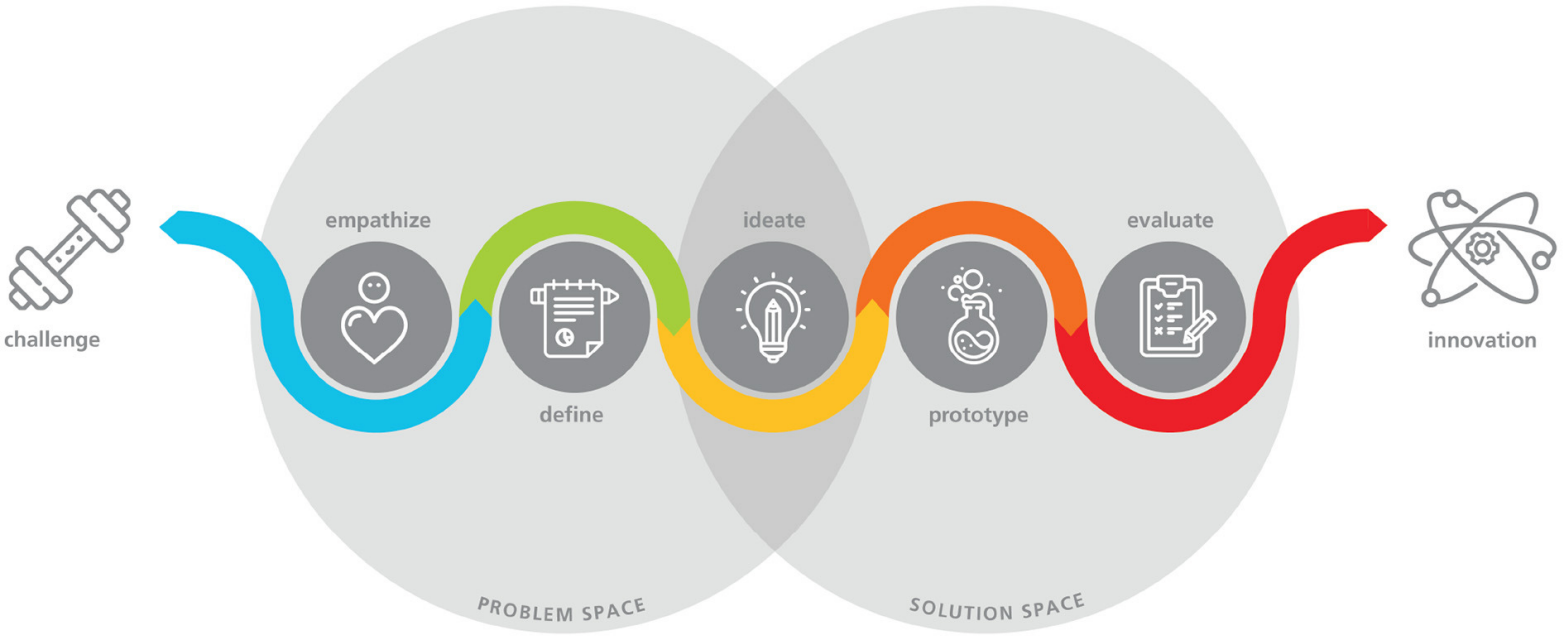
Design thinking process by Fraunhofer FIT (FIT, 2019).

DT models usually follow a multi-stage process, in which activities fall into different categories like exploration, ideation, and materialization. For the purpose of this work, we follow a five step model, for instance as in FIT (2019). The five steps entail: *Empathize, define, ideate, prototype* and *evaluate*. Figure 1 illustrates the iterative DT process. First, *empathize* concerns activities that help the design thinker to build a deep understanding of their target audience and their real-life problems through interviews, desk research, or observation. The gathered information is subsequently structured in the *define* stage through thematic analysis, persona building, or the definition of an actionable problem statements. *Ideation* concerns activities that support the exploration of novel solutions through various brain-storming techniques. Their subsequent incarnation, usually as low-fidelity prototypes that become more sophisticated over time, is carried out in the *prototype* stage. In *evaluate*, design thinkers gather feedback for the created prototypes. As indicated in the figure, DT activities seldom follow each other in a linear fashion. They are rather applied based on their situational necessity.

Examples of the successful application of DT are abundant. In “Creative Confidence,” Kelley and Kelley (2013) describe how DT has helped in the design of MRI machines for the pediatric station of a hospital in the USA. Due to children's nervousness and anxiety of the sterile, small, and noisy tubes it can be difficult for radiologists to get a readable image. By methods of observation, interviewing, and iterative prototyping, the design thinkers could develop a redesigned MRI machine and an accompanying room concept that was not met with fear or nervousness by the patients, for instance a pirate ship-themed MRI room. Hehn and Uebernickel (2018) describe how DT can be used for requirements engineering. In their paper, they have analyzed projects from a data base of a Swiss-German consultancy, which were carried out following a DT approach. *Project Falcon*, for instance, was conducted over the course of 20 months by an interdisciplinary team that was comprised of domain experts, designers and business modeling experts to accommodate for feasibility, desirability and viability of the final product. Activities out throughout the project entailed interviews, persona building, mapping out customer journeys, focus groups, prototyping of mock-ups, user tests, and development of the software.

The concept of co-creation is essential to Design Thinking (Plattner et al., 2012). Consequently, a lot of design-thinkers' work happens in collaborative settings (Kress and Schar, 2012). Design happens as a conversation “[…] with the problem that is being addressed, with materials and artifacts, with our colleagues and with ourselves” (Sirkin et al., 2012, p. 173). While often not stated explicitly, the *DT workshop* in teams of three to five team members can be found in the literature as the preferred modus operandi of co-creative DT activities (see e.g., Brown, 2008; Levy and Huli, 2019; Schwemmle et al., 2021). We attribute this to at least three factors: One being that a workshop can provide a safe space for the participants which can help spark creativity (Daniel, 2020). The second is that workshops usually yield a specific outcome and high output–something that is highly valued in DT (Mueller-Roterberg, 2018). The third factor that a DT workshop can be found especially in scientific literature is that it is a thankful object of study for researchers. Especially in ethnographic studies, individual and group behavior as well as the artifacts that result from the workshops can be observed and studied quite easily (e.g., in Levy and Huli, 2019). Over the course of this paper, we therefore focus on interactions within DT activities such as DT workshops.

### 2.2. Theory of distributed cognition

The theory of DCog was developed by Edwin Hutchins in the 1980's and 90's (Hutchins, 1995a). It is an extension to classic Cognition Theory, in that it sees cognitive processes not limited to the individuals' brains, but rather as being distributed in socio-cultural systems. By this token, the processing and the execution of cognitive tasks take place in the interaction and coordination between individuals and their environment, rather than isolated in an individual brain (Zhang and Norman, 1994; Hutchins, 1995a). With this, Hutchins and his colleagues expand the “boundaries of the units of analysis” of cognition research beyond the individual, and toward “the functional relationships of elements that participate together in the process” (Hollan et al., 2000, p. 175). Cognitive processes can hereby be distributed *between members* of a group, between *internal* and *external* representations, and through *time* (Hollan et al., 2000).

In his works, Hutchins studied so called *systems of cooperation*. He identified several artifacts people use to solve complex tasks. In commercial airline flights, for instance, flight crews need to coordinate their tasks and cooperate in order to successfully execute the flight plan. Hutchins propagates that the expertise to fulfill this task resides not only in one individual crew member, but also in the organization of the tools that the crew members use to solve this task. Thus, no single individual can be attributed to being the actual problem solver. Instead, the complex interplay of many actors and artifacts contribute to the successful achievement of the system's goal (Hutchins, 1995b).

To enhance the understanding and analysis of DCog within small teams, Blandford and Furniss (2005) have developed Distributed Cognition for Teamwork (DiCoT) as a method of analysis. They divide it into the three themes: *physical layout*, which focuses on a physical as well as virtual spaces and its objects, *information flow*, which mainly takes the movement and transformation into account and lastly, *artifacts*. As DCog does not focus solely on one individual, but rather “a collection of individuals and artifacts and their relations to each other in a particular work practice” (Rogers and Ellis, 1994, p. 123), the DiCoT framework takes these differences into account. Thus, far DiCoT has mostly been applied in critical systems in a real environment, e.g., in an emergency medical dispatch (Furniss and Blandford, 2006).

## 3. Method

First, to develop an understanding of current findings on analog and remote DT and according practices, we examined the relevant literature in the research fields of DT and Remote Facilitation of DT. For this, we applied an approach adapted from Mayring's qualitative content analysis (Mayring, 2021). We started by conducting a Google Scholar search with a subsequent snowballing of the references that could be found in the available literature. We chose Google Scholar as the preferred database, because it covers a wide range of material, including scientific papers, gray literature, technical reports, and conference proceedings. We applied the search terms “Design Thinking,” “Remote Design Thinking,” and “Distributed Design Thinking.” We identified 19 articles of potential relevance, 12 of which were journal articles or conference proceedings, six book chapters and one extended abstract. These documents were then distributed among the authors.

Next, each of the authors highlighted important text segments from the selected papers individually, after which the highlighted segments were collected and their main statement was extracted. For instance, the text segment:

“*This means that a participant in a workshop can no longer be considered the “victim” of the spatial planning by an architect or interior designer. She rather becomes co-creator of the space through her interaction with spatial elements. If people involved in Design Thinking realize this shift of power and the active role they can take, it allows them to move from accepting space as-is to changing or even preparing a space based on what best suits their requirements.”* (Schwemmle et al., 2021, p. 125)

was condensed to “*Participants have agency in actively shaping the space.”* In total, 114 of such text segments were highlighted and summarized. Next, each segment was categorized either into *analog* or *remote*, depending on whether they related to analog or remote DT practices. Based on these two categories, we then derived key themes of analog and remote DT practices. With this, we identified the four themes: *Creative Collaboration, Space, Artifacts*, and *Information Management*. Next, we described those four themes with regard to analog and remote DT practices, the results of which can be found in chapter 4.1.

As we have alluded to earlier, DT usually takes place in small teams: “Team-based working modes are an integral part of Design Thinking. Those teams, especially in corporate environments, are increasingly distributed between locations over the globe” (Wenzel et al., 2016, p. 15). Team members of the design thinking team are therefore affected by the concept Distributed cognition when working on complex tasks in the design thinking process. This applies e.g., for working with artifacts: “Throughout the design-thinking process, the team produces several tangible artifacts: empathy maps, journey maps, storyboards, and wireframes, to name a few.” A concept that is known as *representations* in DCog (Hollan et al., 2000; Gibbons, 2016). Hence, in the next step of this paper we aim to deeper understand and learn what DCog might provide to deeper understand the identified themes of remote and analog DT. For this, we drew on the DiCoT framework put forward by Furniss and Blandford (2010), as it is especially suited to facilitate the application of DCog theory on teamwork settings. We then took the categories that we inferred from the literature review of DT and compared them side by side to the superordinate categories (and their respective principles) we found in Furniss and Blandford (2010). We applied the DiCoT framework as a lens to look at the results documented in the spreadsheet, described possible critical elements that could occur when practicing remote DT. The results are described in chapter 4.2. From this, we lastly derived implications for practice when conducting DT activities remotely as opposed to analog.

## 4. Results

In this section we first describe the themes we found when analyzing DT literature and present differences between remote and analog DT practices for each theme. Further, we continue by applying the theory of Distributed Cognition and more specifically the DiCoT framework, to understand and frame these differences from a DCog perspective.

### 4.1. Results of literature review on design thinking

The analysis of the literature yielded four themes of analog and remote DT practices, namely *creative collaboration, space, artifacts*, and *information management*. The following sections describe the findings by juxtaposing practices applied in analogous settings with practices from digital settings. Table 1 summarizes the key findings. Especially the past 2 years have proven to be important as remote work experienced a huge surge due to the COVID-19 pandemic.

**Table 1:** Key results of literature review for remote and analog Design Thinking.

**Theme**	**Remote**	**Analog**
Creative Collaboration	• The quality of creative collaboration is highly influenced by the attributes of the tools that mediate it. • Low costs due to less traveling and less used-up material. • Potentially higher retention rates due to less effort to participate. • Improved scalability of workshops. • Online collaboration tools like digital whiteboards are still inaccessible for large, mainstream cohorts.	• Collaboration extends mono-disciplinary rationales. • It is highly influenced by the space that surrounds people. • Creative collaboration can foster a feeling of ownership and defensive behavior with respect to ideas or the space.
Space	• Digital tool is essential part of the team's success. • They have no physical boundaries. • Extended “space” by enable external cues from the internet. • Asynchronous and synchronous work possible • Meetings have to be scheduled, and are. cannot happen spontaneous.	• Physical room with physical elements (chairs, whiteboards etc.) allows for spontaneous creation of sub-spaces. • People “create” the room in a reciprocal interaction. • Physical space can become a ‘home'.
Artifacts	• Digital artifacts lack “materiality.” • They lose their physical restrictions and their functional qualities with respect to their material attributes. • Digital artifacts can become “smart” through AI (e.g., in *situated agents*).	• Designers transform ideas into “tangible representatives,” i.e., artifacts. • They facilitate communication and collaboration internally and externally. • Artifacts can guide reflective behavior.
Information Management	• Easy to access information through the internet or knowledge management tools. • Asynchronous work is good for information gathering. • Synchronous work enables discussing the value of the gathered information.	• Mostly synchronous work. • Information needs to explicitly made accessible and reproducible. • All information at hand/in the room

#### 4.1.1. Creative collaboration

##### 4.1.1.1. Analog

Creative collaboration between team members is at the heart of DT. Creative collaboration can be influenced by intentionally composing the DT teams with members from different disciplines and cultural backgrounds. Due to its strong emphasis on team-based learning, DT helps to “extend mono-disciplinary rationales by offering a flexible meta-rationale, which counters the restriction of admissible questions or analytical schemes typical of mono-disciplinary thinking” (Lindberg et al., 2010, p. 35).

Collaboration and space are tightly connected with each other. According to Schwemmle et al. (2021), collaborative teams 'create' the space around them, not just by replacing and altering the elements in the space. Rather, the team members construe meaning to the space through interaction with and perception of the space. In this vein, the interaction of individual team members with the space is perceived, either directly or indirectly, by other team members, which influences the behavior and therefore the interaction of those other team members with the space. This type of reciprocal relationship between individuals, space, and teams can lead the team to perceive a space as 'their space' or 'their home', which might provoke a feeling of territoriality (Brown et al., 2005) and, in extension, a sentiment of psychological ownership for the space (as in Dawkins et al., 2017). By distributing and placing certain elements in the space (e.g., chairs, tables, whiteboards, etc.), DT facilitators are able to determine the character of the collaboration between the team members. That way, a team can be prompted to work in a self-organized manner, instead of a hierarchical task distribution. In addition, the creation of a social (sub-)space might foster a positive atmosphere and provide them with a safe-space where they can nurture their personal relationships, rather than work on a specific task (Schwemmle et al., 2021). This phenomenon not just holds true for the concept of space, but also for ideas (Elsbach and Flynn, 2013) where individuals feel like they “own” ideas and show competitive of defensive behavior as part of the “possessive self” (De Dreu and Van Knippenberg, 2005).

##### 4.1.1.2. Remote

Creative collaboration in remote settings is largely influenced by tools that mediate communication. Luther and Bruckman (2008, p. 343) define online creative collaboration as “[…] comprising two key properties. First, people communicate and meet each other chiefly *via* computer-mediated communication. Second, they do so with the purpose of working together to create new artifacts.” Donaldson et al. (2021) describe several benefits of remote collaboration over an analogous setting, like lower costs due to decreased traveling and used up material, potentially higher retention rates due to less effort for the team members to attend, and better scalability of workshops due to the lack of spacial restrictions like room sizes. However, according to Vallis and Redmond (2021), there is a persisting inaccessibility of digital whiteboards and drawing tools to large mainstream cohorts, which leads to the exclusion of certain populations from the design process.

#### 4.1.2. Space

##### 4.1.2.1. Analog

For DT practices in an analog setting, it is important to look at the role a physical space plays for the applied practices. The physical space in which a DT workshop takes place is filled with elements such as seating opportunities, tables, flip charts or whiteboards and other physical elements to work with (Schwemmle et al., 2021). Especially when working together collaboratively on certain tasks people gather around whiteboards or create sub-spaces within a room with portable walls. But not only is space important because of its physical elements “such as its floor plan, distances, or atmospheric cues as perceived by the user (perceived constructed space)” but it is also defined through what people bring with them “such as the individual's perception, its experiences and resulting behaviors (reflected constructed space)” (Schwemmle et al., 2021, p.125). Because space is constantly changing throughout the process of iterative work, it must be understood on a behavioral level as well (Brown, 2008). People in a designated space are not merely caged in it, but they rather “create” space by interacting with the physical elements in it and assigning meaning to these elements or their arrangement. The physical space influences people but can also be influenced by them (Sirkin, 2011; Schwemmle et al., 2021), thus forming a reciprocal relationship as touched upon earlier. When it comes to the atmospheric perception of space, for DT practices it is necessary to let people feel like they have a *home*. This helps to foster co-creation and inspiration as people feel that a certain space is “their own” which “creates safety for a team, allows identification and fosters well-being” (Schwemmle et al., 2021, p.133).

##### 4.1.2.2. Remote

In remote DT a variety of digital tools can be used that set up and guide the scenery of DT activities. They provide a digital space where a physical space is not available. This requires having a tool to communicate during the workshop and a tool to collaborate in a virtual space (Sirkin, 2011). Wenzel et al. (2016, p. 16) emphasize that “the digital tool is not only a plain functional instrument. In fact, with its usability and acceptance it is a relevant factor for the teams' success. Thus, making the tool an important player among the members of a team requires its interplay with the team's working situation.” In consequence, a virtual space where teams can coordinate and carry out their work and collaborate on specific tasks and design a solution is an essential part of the remote DT process. Additionally, a virtual space has no physical boundaries. Especially when it comes to innovative thinking and the creation of ideas, extending ones space beyond the immediate surroundings using digital tools might offer a wider perspective and inspiration (Unger et al., 2021). Team members can access external cues such as images or information *via* search engines and extend their knowledge (Vallis and Redmond, 2021).

Along with the workshop design that might integrate single person work time or might even schedule the process within an extended period of time, a virtual space holds the chance to work on a personal scale and speed and at the same time synchronize the teams' progress, which is a challenge to master (Yarmand et al., 2021). Asynchronous work however, might lower hesitations in creative thinking such as the fear of speaking up, etc. Nonetheless, a virtual space is a challenge when it comes to spontaneous collaboration and might even hinder team members to get help in the moment they really need it (Sirkin, 2011).

#### 4.1.3. Artifacts

##### 4.1.3.1. Analog

The creation of and interaction with artifacts is crucial to DT: “Design-as-practice cannot conceive of designing (the verb) without the artifacts that are created and used by the bodies and minds of people doing designing” (Kimbell, 2012, p. 135). Designers transform ideas into tangible representatives (i.e., artifacts), which not only facilitate communication and collaboration within the team, but also with external stakeholders and help the designers to stay in touch with the problem-relevant environment (Lindberg et al., 2010). For Jung and Stolterman (2010, p. 153), “design can be considered as a process of creating meaning with proper materials based on exploratory practice with them,” thus highlighting the importance of physicality and tangibility of artifacts in DT. Ghajargar and Wiberg (2018, p. 5) describe the potential for artifacts to guide reflective behavior, with “‘reflection' referring to the action of reflecting on information provided, and being informed about the consequences of an action or behavior and to create puzzling and surprising effects”. As physical representations of an idea, artifacts as prototypes help designers not just to explore their solutions, but also to communicate these solutions with the outside world. These types of artifacts, however, do not only have a profound influence on the actors in a design process—they also play an important role in research. In design research and HCI the *research through design* approach lets researchers examine problem spaces and create solutions “through the construction of artifacts” by applying methods informed by the work of designers (Zimmerman and Forlizzi, 2008, p. 42).

##### 4.1.3.2. Remote

Due to their non-physicality and non-tangibility, digital artifacts play somewhat of a different role in DT compared to their analog counterparts. According to Balters et al. (2021, p. 10f), “[t]he use of artifacts (analog or digital) affects practically every facet of the DT methodology and practice. The absence or curtailment of artifact usage and accessibility combined with the absence of face to-face interaction together severely changes the quality of interaction and outcome between people during an innovation event.” On the one hand, as digitization progresses, artifacts (such as prototypes or work tools) lose their materiality, or become *materials without qualities*, as Löwgren and Stolterman (2004) put it. Thus, artifacts lose their physical restrictions and their functional qualities with respect to their material attributes. This becomes especially interesting if we consider spatial artifacts as tools for the design process, like the properties of a room or the furniture in it. Examining this loss of material quality becomes increasingly important, as more and more collaborative design happens digitally and artifacts become digitized. On the other hand, artifacts gain certain attributes as well, for instance, as tools are becoming increasingly smart. AI-supported tools, so called *situated, reactive* or *behavioral agents* are context aware, proactive (i.e., can act autonomously), preemptive (i.e., help humans to prevent errors) and interactive (Ghajargar and Wiberg, 2018).

#### 4.1.4. Information management

##### 4.1.4.1. Analog

Information gathering and processing in DT activities are important factors to successfully running for example DT workshops. This counts for existing knowledge as input for and output of these activities as well. Tracing information about design decisions throughout all phases of the DT process is therefore key for iterative work. If information gets lost “the evaluation of ideas is often restricted to the prototype alone and cannot be navigated back to the original source of the design decisions” (Gabrysiak et al., 2011, p. 221). Analog group work is mostly synchronous, all the necessary information is either contributed by the DT facilitator or by the team members themselves (Schwemmle et al., 2021). It is therefore important that all information in the room of the DT workshop is accessible as well as reproducible at any time (Gabrysiak et al., 2011). When communicating the result of a DT activity, a tested prototype for e.g., a product, the engineering team has to be aware of design decisions made so they can take them into account in their work. Information not transferred might lead to the end product not being in line with the envisioned idea of the DT Team.

##### 4.1.4.2. Remote

Remotely working in the DT phases on the one hand makes it easy to access information, e.g., by searching for additional information online and by using the landscape of free digital tools that support design processes. This might help bringing everyone on board, “when information has to be shared among all participants–for instance, during the welcome phase of a workshop, introducing the challenge or giving interim inputs, and, finally, to present results to all participants and maybe even to an external audience” (Schwemmle et al., 2021, p. 129). On the other hand a challenge in a remote setting is to develop a common knowledge base. Here, working asynchronously for getting more information but synchronously for discussing the value of it might be necessary.

### 4.2. Application of DiCoT on DT

After having identified four themes in the DT literature that highlight the differences between remote and analog DT practices, we aim at understanding the implications of the ongoing digitization on those practices. For this purpose, we use DiCoT as a framework to look at our findings from a DCog's perspective. DiCoT has been applied as an analytical framework for collaborative work settings before (e.g., in Hussain and Weibel 2016). Given the highly collaborative nature of DT, we consider this framework particularly suited for helping us uncover why the differences between analog and remote DT occur. Blandford and Furniss (2005) describe their themes *physical layout, information flow* and *artifacts*. Each of their themes is further divided into different *principles*, which we will consider in the context of DT in the following.

#### 4.2.1. Physical layout

The *physical layout* includes all the environmental aspects influencing the performance of the cognitive system. Those environmental aspects may refer to auditive, visual, or tactile stimuli, which shape the perceptions of humans and thus have a direct impact on their computational capabilities.

When it comes to remote DT practices *space and cognition*, which are one of the principles of the physical layout, differ from an analog setting. In the analog world, a table can be moved and stacked with papers, thus help people to reduce complexity and make choices. In the remote setting space is effectively infinite and not limited to borders e.g., of a table. Artifacts that are out of sight cease to exist, which might increase the complexity of collaboration. Also, digital representations of information are not easily perceived as natural (*naturalness principle*). Even though digital tools try to copy the real world whenever possible, e.g., a sticky note in real life and its representation in an online tool are much alike, it is especially the interaction with digital representations which differs from the interaction with analog ones. This might provide more effort for mental transformations to make use of those representations.

Furthermore, tools for remote collaborative work can provide assistance when prioritizing content or tasks (e.g., the “bring everyone to me”-function, or the mini-map in the collaborative online tool Miro), which in the DiCot framework can be found in the *perceptual principle*.

The principle of *situation awareness* describes that people need to be informed about details of a situation such as what is planned and what is going on. Blandford and Furniss (2005, p. 29) even state: “The quality of this situation awareness can be influenced by how accessible the work of the team is.” In the analog context the proximity toward the team is important, which means one can observe or even overhear what is happening. In a remote setting there is a lack of proximity which might lead to less situation awareness. The digital landscape of supporting tools for remote work also provide the opportunity to get a better overview on what other teams work on. Especially for interdisciplinary teams working globally this might create perceived proximity to other teams. This also holds for the *horizon of observation* which provides the team member with an overview of everything that can be seen and heard. Even though being present in one physical space helps by focusing on the environment, a remote access to all information and digital possibilities of documentation such as video recording or chatting might broaden the horizon of observation as it can be accessed any time and from everywhere. However, this requires a moderation that focuses on a common understanding of the teams' horizon of observation. Being aware of ones surroundings also includes the *arrangement of equipment*, which is key to processing information. Here, an analog setting is limited to the space and equipment at hand, which might be of advantage when focusing on reducing complexity of problems. In a remote setting, space and access to information is not limited due to a broad range of digital tools. On the one hand, this might help to understand complex problems and get inspired more thoroughly, but on the other hand could lead to an overload of information that cannot be processed anymore.

The last principle as part of the physical layout is *subtle bodily supports*, which mostly comes into effect in analog DT so far, e.g., when team members can point at things and speak with their body. This is limited in a remote setting as it is not the finger as part of the body pointing on things, but its digital representation, e.g., a cursor (Sirkin, 2011).

#### 4.2.2. Information flow

Information flow revolves around the interaction of entities within the cognitive system. This may include the communication between team members and the transformation of information or tools that facilitate the information flow. Looking at a remote DT workshop representations (i.e., physical realizations of artifacts) are different from their analog counterparts. This has consequences for the information management and therefore flow of information.

First, the principle of *information movement* seems to be present differently within the analog than the remote setting. Information in the analog setting can be, for example, “passing physical artifacts; text; graphical representation; verbal; facial expression; telephone; electronic mail; and alarms” (Blandford and Furniss, 2005, p. 32). In the remote setting, information is mostly provided in a two-dimensional way, e.g., an emoji on an online whiteboard. Both, analog and remote DT activities support information movement. Still, being online might put a different kind of speed and complexity on information movement, as much information can be accessed quickly and with low effort, for instance because it easier to copy and paste information like pieces of text, from A to B.

The principle *transformation of information* describes changes in the representation of information. One example is filtering information, which in the DT process is an essential part. Several artifacts, like sticky notes, are gathered, sifted and structured and thus distilled to one key aspect which is written on a new sticky note (so called *clustering* or *thematic analysis*). The transformation of information can therefore be realized in both, the analog and remote DT process. Some digital tools for remote work even feature tracing prior steps within the process and therefore allow asynchronous collaboration.

An important activity of DT is to channel all information that is necessary to develop innovations, whether it is in a physical or a digital space, so that every team member has access to it at any time from anywhere. The principle of *information hubs* describes that information from different sources are brought and processed together. Especially when a team makes decisions an information hub helps to define further steps. Elements in a physical room (e.g., whiteboards or flip charts) as well as in a digital room (e.g., online whiteboards) can support this decision-making process by displaying all information in one place and therefore promote effective work. However, the distribution of information may differ in its extent and the ways it is provided. When a lot of information is shared at the same time, the principle of *buffering* applies, which aims at withholding information until it can be introduced without the risk of errors or conflicts with ongoing activities.

#### 4.2.3. Artifacts

The third topic centers around artifacts and how their design enhances cognition of the individuals within the system. This includes the layout of an artifact, for instance the form of a sticky note, as well as the physical movement of it, e.g., hanging a sticky note on a chart or moving it. The principle of *mediating artifacts*, for instance, describes those types of artifacts that help the team to complete the task. In remote DT, digital artifacts have different attributes as compared to haptic ones. For example, a lot of people's interactions with artifacts relate back to their positioning in their immediate environment. That means that artifacts can be used to create *scaffolding*—the second principle of artifacts—for example by placing a marker where a task was left and is to be picked up again in the future. In digital spaces, however, Design Thinkers might lose the perception of an artifact's location due to the space's lack of physical boundaries. Also, as alluded to earlier, materiality plays a key role in people's interaction with those artifacts. The absence of the tangibility of a sticky note, for instance, could make it harder for team members to use these artifacts to their advantage (like passing a sticky note to another team member). Artifacts also serve a representational function in that they create *goal parity*—the third principle of artifacts—between the actors involved. Prototypes, for instance, mediate between the contemporary and a desired future state and communicate a DT teams vision and thought process to people external to the team. Digital prototypes, however, do not convey the same type of experience as haptic ones do. Practitioners should therefore pay close attention to the way they create and use artifacts. Given that digital prototypes are for now impossible to touch, smell, or taste, practitioners have to rely on visual and auditive clues for their target audience to represent the desired future state. They should therefore rely on methods like storytelling or scribbling. Additionally, they should pay attention to the arrangement of artifacts on the digital spaces that they are working on. If too many digital artifacts occupy the digital space and get pushed outward, or if too many digital spaces are created, people might lose awareness about their existence.

## 5. Discussion

### 5.1. Implications for practitioners

After having elaborated on the perspective of DCog analog and remote DT practices through the lens of the DiCoT framework, we now address specific implications for DT practitioners when applying analog and remote DT. As the ongoing digitization requests applying both, analog and remote DT, differences and how they can be used efficiently have to be considered when designing DT workshops.

As mentioned above, *physical layouts* in remote settings differ from analogs ones. Space is virtually infinite, which can lead to a feeling of being lost or overwhelmed. Practitioners should use tools that allow them to structure the space into working areas and assign themes to these working areas. This could help participants of a DT workshop to better orient and reduce the complexity of the virtual space. Functions like “Bring to me,” where the participants' view on a digital whiteboard is pulled to the facilitator, can also help with streamlining attention. In order to create a feeling of naturalness, practitioners should use tools that allow them to create items that represent a natural form, like a digital sticky note. They should also point out their natural representation when they introduce the tools to inexperienced workshop participants. This can give them a cognitive aid and make it more effortless for them to use these items and to feel at ease while using them. The size of the items (e.g., a sticky note) used in the DT process can also be utilized by Design Thinkers. Increasing the size of an item may indicate that it is of higher importance than others. The same holds true for the color or the shape of an item. However, in some situations this could be counterproductive. In brainstormings, for instance, all ideas should initially have equal weight and importance. If certain ideas are displayed on a larger sticky note, this could lead to a selective perception (Pronin, 2007) in the further progress. In general, situational awareness is harder to convey in remote settings. Hence, practitioners should be much more descriptive in their language and explicate most of what they are currently doing verbally. When conducting workshops with inexperienced Design Thinkers, this needs to be pointed out regularly, as it creates transparency and awareness for the other team members. Additionally, enabling participants to independently move through spaces or rooms (e.g., break-out rooms) promotes autonomy. Generally, tools like digital whiteboards or video communication should be kept as simple as possible, as switching between tools demands cognitive resources from the participants (Skulmowski and Xu, 2022).

To ensure good *information flow*, practitioners should explicate verbally when they move a digital item from one point to the other. Team members' attention can be focused on a different part of the working area and they might not even be aware, that an item was moved. Generally, for team members who are not proficient with tools like digital whiteboards, moving items might not be an easy task. Warm-up games in the beginning of a workshop could help to practice this. A big advantage of digital items is that they can be copied and pasted virtually without effort–other than analogous ones. By copying and pasting created items to other sections of the working area, rather than moving them, the entire process becomes better traceable. Also, by clustering certain information together in information hubs and giving these hubs prominent thematic captions, it can help practitioners to find information quicker and easier. One of these information hubs could be an *Idea Parking Lot*, where team members can “park” their spontaneous ideas for later reuse. Creating spaces where information can be “parked” might help the team to reduce cognitive capacities and therefore focus on the information at hand. Yet, information is not lost but can be introduced at a later stage.

*Artifacts*, as the tools that help DT practitioners to generate and convey their ideas, can also be used to create structure. Some tools are better developed to create ideas, while others might be better suited for prototyping. Generally, practitioners should think about the purpose at hand prior to the DT activity and then chose a fitting tool. Also, artifacts can create structure, in that they can indicate to the practitioners where in the DT process they currently located, for instance through a Kanban board or other visual cues. Additionally, to make up for the lack of materiality of artifacts like prototypes, practitioners could break through the two-dimensionality of virtual artifacts, for instance by printing out prototypes of a web-page or by providing team members with do-it-yourself kits like Lego Serious-Play, in order for them to rebuild the prototype in real-life.

### 5.2. Future outlook and AI technologies

Finally, as practitioners in the field of computer science we deem technologies like AI to potentially contribute to the future of DT practices. In the following we therefore want to offer our thoughts what AI might hold for DT practices in the future.

Especially remote DT activities hold much potential for incorporating AI to enhance workshop and learning experiences. For instance, Eve, a software tool that helps designers to create low-fidelity prototypes and transition them into mid-fidelity to high-fidelity prototypes *via* machine learning was presented by Suleri et al. (2019). Such a tool can help design thinkers, not just to learn how to prototype faster and easier, but also to enhance cognition by distributing difficult tasks to the software tool. Another possibility, provided by the use of AI within the physical layout, lies within the arrangement of equipment. Being aware of ones surroundings and the equipment at hand might be supported by AI. For example, an AI-based companion could help a facilitator to raise awareness of available artifacts and could further accompany creative processes by using those artifacts (Verganti et al., 2020). This could also trigger behavior change, as facilitators with lower experience have a higher level of guidance, thus can implement new methods more easily.

For the *information flow*, AI could support the transformation of information, by offering intelligent clustering or filtering (Verganti et al., 2020). This means, the decision, whether an information is important now or if it can be hold up until later, called buffering, which currently has to be made by a human intelligence could also be assisted by AI. In addition, AI could be supportive in making those choices for example with recommender systems. Further, AI algorithms could also highlight the most important information to allow information hubs.

*Artifacts* can already be supported by AI, e.g., with the “stickies capture” in Miro (smart text recognition), where analog sticky notes are automatically recognized and digitized. Furthermore, the creation of artifacts could also be supported by such AI powered tools, e.g., algorithms that could be included in brainstorming activities, or while gathering information on target groups. This could support facilitators, as well as team members of DT activities.

Participants of DT activities need to be able to trace where, e.g., information for personas are coming from, which might help to minimize the risk of research biases. It is therefore essential that algorithms, especially those that support decision making, are transparent and explainable (Explainable AI; XAI) (Gunning et al., 2019).

### 5.3. Limitations

This work has been conducted from the perspective of DT facilitators. For future research, the perspective of team members of DT activities such as workshops should be taken into account to broaden the significance of the results for the respective target groups. This paper aims at providing a DT facilitators view on the changing environment, that has been caused by the COVID-19 pandemic, rather than empirical insights. Thus, collecting primary data could yield further insights to practitioner guidelines and improve the overall experience of team members and facilitators in creative work. This may also foster insights into team members' appropriation of digital tools in DT activities and how this might influence their practice.

Due to the COVID-19 pandemic, the demand for digital tools has increased, as many companies adopted hybrid working modes and therefore needed more and more sophisticated tools to facilitate this change. This has accelerated the development and improvement of various digital tools for remote collaboration, like digital whiteboards or video conferencing tools. Due to the high and increasing demand, these tools grow rapidly in functionality, with providers adding more and more features to their products, rendering remote collaboration easier in some aspects (e.g., asynchronous work) and more complex in others (e.g., efficient communication). As this is a continuous process, publications differ in the functionality in the respective tool that has been used, which may have an impact on the user experience and feasibility of workshops for practitioners. As the pandemic forced many practitioners to quickly switch to a remote environment on a day-to-day basis, recent reflective insights on findings for remote DT might not have been published yet at the time of this paper.

This work aimed at looking at the DT process as a whole and not divide it into its five phases. For future research, it may be of interest to examine how the different phases are executed in analog and remote settings and where they differ most with regards to interaction and distributed work. This would be an even closer look into DT activities and might allow for more precise practitioner guidelines. Even though there is a lot potential for the integration of AI into DT practices, it also holds risks as well. These may include increasing the cognitive load (Skulmowski and Xu, 2022) and no sufficient technical competency by team members and facilitators.

## 6. Conclusion

We have pointed to the potential influence that digitization and the development toward increased remote-based work might have on DT practices. We have analyzed scientific literature from the relevant research areas and identified four themes in which remote DT practices might differ from analog ones. We have used the theory of DCog and the DiCoT framework according to Blandford and Furniss (2005) to put our findings in perspective and collect concrete notes for DT practitioners when conducting remote DT workshops. Interactions with digital tools have had an increased importance in many workplaces, which allows us to draw the connection to the theory of DCog. Therefore, a new light has been shed on the relevance of corresponding theories and concepts. This allows future research on remote work settings to take DCog closer into account to evaluate digital tools and interactions with those.

## Author contributions

With his expertise in Design Thinking and HCI, DW contributed substantially to the introduction and the literature review of Design Thinking literature, and contributed parts of the section on Distributed Cognition. With her expertise in Human-Centered Design and Design Thinking, ES contributed substantially to the literature review of Design Thinking literature, designed and drafted (Table 1), and contributed to the limitations section. With her expertise in psychology and computer-supported collaborative learning, A-KB contributed substantially to the literature review of Distributed Cognition, and the conclusion and limitations sections. With his Ph.D. in computer science and his experience as the head of department for Human-Centered Engineering and Design at Fraunhofer FIT, RR contributed to the conclusion and future directions of the paper. All authors contributed to editorial tasks in all chapters.

## Funding

The research leading to these results has received funding from the European HORIZON 2020 program under grant agreement no 857202, the DEMETER project.

## Conflict of interest

The authors declare that the research was conducted in the absence of any commercial or financial relationships that could be construed as a potential conflict of interest.

## Publisher's note

All claims expressed in this article are solely those of the authors and do not necessarily represent those of their affiliated organizations, or those of the publisher, the editors and the reviewers. Any product that may be evaluated in this article, or claim that may be made by its manufacturer, is not guaranteed or endorsed by the publisher.
